# Exposure to power frequency electric fields and the risk of childhood cancer in the UK

**DOI:** 10.1038/sj.bjc.6600602

**Published:** 2002-11-12

**Authors:** J Skinner, T J Mee, R P Blackwell, M P Maslanyj, J Simpson, S G Allen, N E Day

**Affiliations:** Strangeways Research Laboratory, University of Cambridge, Wort's Causeway, Cambridge CB1 8RN, UK; National Radiological Protection Board, Chilton, Oxfordshire OX11 0RQ, UK; Leukaemia Fund Research Centre for Clinical Epidemiology, University of Leeds, 30 Hyde Terrace, Leeds LS2 9LN, UK

**Keywords:** electric fields, childhood cancer, leukaemia

## Abstract

The United Kingdom Childhood Cancer Study, a population-based case–control study covering the whole of Great Britain, incorporated a pilot study measuring electric fields. Measurements were made in the homes of 473 children who were diagnosed with a malignant neoplasm between 1992 and 1996 and who were aged 0–14 at diagnosis, together with 453 controls matched on age, sex and geographical location. Exposure assessments comprised resultant spot measurements in the child's bedroom and the family living-room. Temporal stability of bedroom fields was investigated through continuous logging of the 48-h vertical component at the child's bedside supported by repeat spot measurements. The principal exposure metric used was the mean of the pillow and bed centre measurements. For the 273 cases and 276 controls with fully validated measures, comparing those with a measured electric field exposure ⩾20 V m^−1^ to those in a reference category of exposure <10 V m^−1^, odds ratios of 1.31 (95% confidence interval 0.68–2.54) for acute lymphoblastic leukaemia, 1.32 (95% confidence interval 0.73–2.39) for total leukaemia, 2.12 (95% confidence interval 0.78–5.78) for central nervous system cancers and 1.26 (95% confidence interval 0.77–2.07) for all malignancies were obtained. When considering the 426 cases and 419 controls with no invalid measures, the corresponding odds ratios were 0.86 (95% confidence interval 0.49–1.51) for acute lymphoblastic leukaemia, 0.93 (95% confidence interval 0.56–1.54) for total leukaemia, 1.43 (95% confidence interval 0.68–3.02) for central nervous system cancers and 0.90 (95% confidence interval 0.59–1.35) for all malignancies. With exposure modelled as a continuous variable, odds ratios for an increase in the principal metric of 10 V m^−1^ were close to unity for all disease categories, never differing significantly from one.

*British Journal of Cancer* (2002) **87**, 1257–1266. doi:10.1038/sj.bjc.6600602 www.bjcancer.com

© 2002 Cancer Research UK

## 

The UK Childhood Cancer Study (UKCCS) was a national, population-based case–control study designed to investigate the possible role of several exposures in the aetiology of childhood cancer, through a set of *a priori* hypotheses (UKCCS Investigators, 2000b). One hypothesis related the risk of the development of childhood cancer, particularly leukaemia and brain tumours, to increased exposure to power frequency electromagnetic fields (EMF). In our primary investigation of this hypothesis, we found no association between measured power frequency magnetic fields and risk of childhood leukaemia or any other cancer (UKCCS Investigators, 1999). In a separate analysis of sources of electricity supply near the homes of our subjects, with magnetic field levels calculated from historical load data, we found no evidence that either residential proximity to selected electrical installations or the magnetic field levels they produce in the UK is associated with risk for any malignancy (UKCCS Investigators, 2000a). Here, we report the results of residential electric field (E-field) measurements made on a subset of the study subjects for whom we had magnetic field measurements. The measured electric fields are those in the absence of a person. We also analyse unperturbed electric field strengths from high voltage overhead power lines, calculated for all subjects for whom we had details of sources of electricity supply near the home.

## MATERIALS AND METHODS

### Study participants

Children aged 0–14 years inclusive in England, Scotland and Wales diagnosed with a malignant neoplasm were eligible for inclusion in the UKCCS. We identified cases through collaboration with paediatricians and oncologists. Case accrual began in January 1991 in Scotland and ended in December 1994. In England and Wales, case registration started in April 1992 and finished in December 1994 for solid tumours, December 1995 for non-Hodgkin lymphoma, and December 1996 for leukaemias. All case diagnoses were pathologically reviewed. For each case, two controls were randomly selected, matched on sex and month of birth, from the list of the Family Health Authority or Health Board in which the case lived. Both cases and controls were ineligible if they were born outside Great Britain or had had a prior malignancy. If an eligible control family decided not to participate, another family was approached, until two control families participated. A total of 3838 cases and 7629 controls took part in the study as a whole (UKCCS Investigators, 2000b). For all of the EMF investigations, we used only one of the controls participating in the main study because of limited resources.

We assigned a pseudo-diagnosis date to each control (the date at which the control child was exactly the same age as the corresponding case at diagnosis). To be eligible for magnetic field measurements, each child in a case–control pair must have lived in a single house during the year prior to diagnosis or pseudo-diagnosis and have still been living there at the time of measurement. Children aged less than a year at diagnosis or pseudo-diagnosis must have lived at one address since birth and have still been living there at measurement. We presented results based on magnetic field measurements for 2226 case–control pairs (UKCCS Investigators, 1999). Here, our primary analysis includes electric field measurements for which acceptable instrument function checks were made. These were available for 549 subjects (273 cases and 276 controls). We also examine results for all measurements without invalid checks, which were available for 845 subjects (426 cases and 419 controls).

### Data collection

The UKCCS EMF component was designed primarily to investigate exposure to power frequency magnetic fields. The magnetic field survey was conducted as a two-phase study, with residential measurements taken for all participants in the first phase. More extensive residential measurements were taken in the second phase (phase II) for matched case–control pairs where either of the pair had an average residential exposure estimate in the top 10% of such estimates. Phase II also included subjects living near to certain power lines and other pre-defined electrical facilities, or living in homes where specified appliances were identified. The UKCCS residential electric field pilot study was restricted to phase II of the EMF study, with electric and magnetic field strength measurements taken at the same locations simultaneously. The electric field pilot study did not begin at the same time as magnetic field measurements, but all phase II assessments after a given start date in each UKCCS region included measurements of ELF electric field strength. This gave a pilot study population of 926 individuals.

Single axis measurements of ELF electric field strength were made with a sensor (described below) attached to the commercially available Emdex II magnetic field meter (Enertech Consultants Ltd., Campbell, CA, USA), which operates within the broadband frequency range 40–800 hertz (Hz). The phase II measurements comprised: (a) four 3-min spot measurements taken at the centre of the family room, at the centre of the child's bed, at the centre of the pillow and at the bedside position to be used for the 48-h measurement; (b) a 48-h measurement taken by the side of the middle of the child's bed; (c) a repeat of the four spot measurements after the 48-h measurement. Bed measurements were made with the sensor supported on a short polypropylene pole attached to a 29.5 cm diameter base of the same material, which acted to spread the sensor load. This gave a measurement location 15.5 cm above the centre of base. All other measurements were made using a larger polypropylene stand at 1 m above floor level. During spot measurements, the sensor assembly was orientated for 1-min periods in each of three mutually orthogonal axes by means of locating pins. After each orientation the technician retired from the room. For the 48-h measurement the sensor assembly was left in the vertical orientation. For the spot measurements in the bedroom, appliances and lights were switched on or off to replicate the state in which they were usually left at night, though lights were left on if necessary to see the instruments. Spot measurements in the centre of the family room were made with room lighting and appliances as found. Appliances were switched on and off freely by the family during the 48-h bedside measurement. A sampling interval of 3 s was used for the spot measurements, adjusted to 10 s for the 48-h bedside measurement.

The technicians carrying out electric field assessments could not see the instrument readings and subsequent processing of measurement records was blind to case–control status. Individual measurement records were evaluated visually through the commercially available software accompanying Emdex II meters. Field perturbation by the intervention of the technician allowed segregation of the trace into orthogonal components. Values of the mode (the most frequently observed value) were computed for sections of the record corresponding to measurements of a particular component, to allow visual processing to be checked and revised as necessary. The mode was used because mean values were more susceptible to operator approach if the times between field perturbations had been identified incorrectly. RMS values of electric field strength for the three orthogonal components were root sum of squares (RSS) combined to yield the resultant field strength for each spot measurement location. The 48-h bedside measurements yielded only the vertical component of electric field strength.

### ELF electric field sensor

To measure electric fields, a single-axis displacement current sensor was connected to the commercial Emdex II meter, used in the magnetic field investigation, via the auxiliary socket. The sensor consisted of two metal half-cylinders that enclosed the meter. This was contained within an outer polypropylene cylinder that could be orientated in three mutually perpendicular directions. To improve sensitivity it was necessary to make a small modification to auxiliary input circuitry. The Emdex II instrument was calibrated by injecting a known 50 Hz current into the auxiliary socket and adjusting the input sensitivity to obtain the desired response. The appropriate calibration constant was entered into the software supplied with the meter. The sensor-instrument assembly was tested using a parallel plate system traceable to national standards and found to be accurate and linear over field strengths up to 9 kV m^−1^. On final assembly, each sensor underwent a calibration check in a nominal field of 100 V m^−1^. The resolution of the completed instrument was better than 1 V m^−1^ in the range 0–87 V m^−1^, 1.6 V m^−1^ in the range 56–1390 V m^−1^ and 25 V m^−1^ for the range 1–9 kV m^−1^. The overlapping ranges and differing resolutions are a result of the Emdex II's three automatically selected ranges. Worst-case accuracy for E-field measurements, including the contribution from the Emdex II meter itself, is estimated to be ±20%. Calibrations were performed using the small stand, as used for bed measurements, and the uncertainty in differing orientations of the sensor on the stand have been included in this estimate. For practical reasons, calibrations were not performed using the sensor with the 1 m stand. However, repeat measurements with and without the stand (replaced by low density, low dielectric constant ‘Eccostock SH’ foam) gave identical resultant field levels that were within 4% of field strengths measured by a commercial instrument with calibration traceable to national standards.

Apart from the calibration of the sensor assemblies, a check source was designed and introduced after the study had begun, to assess sensor response over time. It consisted of a pair of electrode plates fed from a low voltage AC supply and arranged to provide capacitive coupling to the sensor. The dimensions of the plates were chosen so that the instrument recorded a nominal field strength value of 100 V m^−1^ though tolerances in construction and coupling gave a range of response from 55 to 165 V m^−1^ across sensor–check source pairings. The check source procedure was carried out before and after each set of residential measurements. Two particular investigator-dependent problems could arise when using the check source: the sensor was not connected to the Emdex II instrument or a recording of ambient field strength was made without the check source energised. Measurement sets attached to check readings outside the above response range were designated invalid. Time series of valid readings from individual sensor–check source pairings showed consistent response, generally within 25% of the mean value. Of all measurements, fewer than 9% were excluded from all analyses on the basis of check source validity.

### Calculated E-fields

In order to identify important electricity sources such as power lines near to homes an external-sources questionnaire was completed for each study subject as part of the main investigation. The questionnaires, completed by NGC and regional electricity company staff, employed voltage and distance criteria of (a) an NGC line within 400 m, (b) an REC line of 66 kV or higher voltage within 200 m, 140 m or 100 m determined by rating and (c) an REC line of 11–33 kV within 80 m (double circuit) or 50 m (single circuit) of a home. For the homes near to power lines it was possible to estimate the unperturbed electric field strength outside the home using the voltage, phase transposition and distance information provided on the questionnaires. National Grid Company's EM2D programme was used to produce a two-dimensional solution for the electric field, allowing where possible for type of tower, type of conductor and conductor clearance. If there was more than one power line near a home, the overall field strength was estimated by RSS-combining the individual line contributions. The error in the computation was estimated to be typically less than ±20%. This arose mainly from the uncertainty attached to the input parameters derived from the questionnaire and the possible departures from a two-dimensional solution.

### Statistical analysis

Our primary results are those for measurements with two valid check readings. We also present results obtained using all measurements without invalid check readings; these include measurements with no check readings and those for which only one check reading was made and this was valid. This increased the sample size at the expense of reliability; around 15% of the additional measurements could be expected to have had invalid check readings (Table 1

**Table 1:**
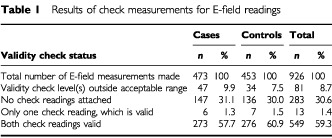
Results of check measurements for E-field readings

).

Risks of acute lymphoblastic leukaemia, all types of leukaemia, cancers of the central nervous system (CNS), other malignant disease and total malignant disease were investigated using two exposure metrics: the mean of the two pairs of spot measurements made on the bed (pillow and bed centre) and the mean of the four pairs of household spot measurement (family room, bedside, pillow and bed centre). The mean of the bed measurements was used as our principal exposure estimate, as it was reasonably certain that the child spent a significant part of his or her time in this location. The mean of the bed measurements was divided into three categories (<10 V m^−1^, 10–<20 V m^−1^, ⩾20 V m^−1^) based on approximations of the median and 90th percentile of the control population. We also modelled exposure as a continuous variable, to investigate the trend in risk with exposure and to avoid the problems inherent in arbitrary cut-offs. We calculated odds ratios for an increase of 10 V m^−1^ in both the mean of the bed spot measurements and the mean of the household spot measurements.

The spot measurements were calculated from the resultant of three sequential measurements of 1-min each in orthogonal directions. The 48-h bedside measurement recorded the vertical E-field component alone. As the resultant is calculated from ✓(*v*^2^+*h_1_*^2^+*h_2_*^2^), estimating *h_1_* and *h_2_* by *v* gives ✓(*v*^2^+*v*^2^+*v*^2^)=*v*✓3. The resultant estimated from the vertical component alone was compared to the actual resultant for the spot measurements made in the 48-h measurement location (Table 2

**Table 2:**
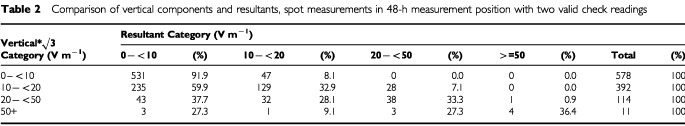
Comparison of vertical components and resultants, spot measurements in 48-h measurement position with two valid check readings

). Table 2 suggested that *v*✓3 was not a good proxy for the resultant, implying that there must be significant variation between the measurements on the three axes. The 48-h measurement was therefore not used in any exposure estimates.

Figure 1

**Figure 1:**
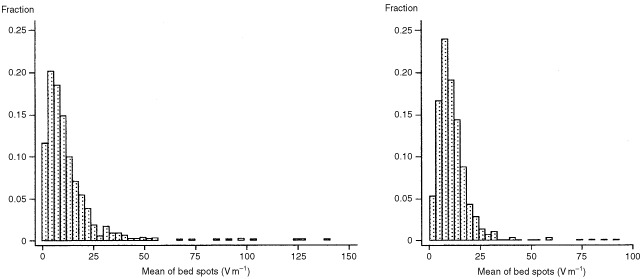
Distribution of mean of bed spots and mean of all spots for E-field readings with two valid check measurements (*n*=549).

shows the distribution of the two exposure metrics used in our analyses: the mean of the bed spots and the mean of all the spot measurements made in a home, for all E-field readings with two valid check measurements. As both display a considerable degree of skewness, all correlations are given on a log scale.

Table 3a

**Table 3a:**
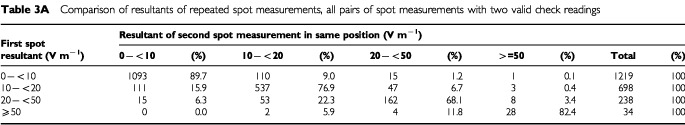
Comparison of resultants of repeated spot measurements, all pairs of spot measurements with two valid check readings

gives a comparison, by category, of spot measurements made in the same position separated by 48 h and Table 3b

**Table 3b:**
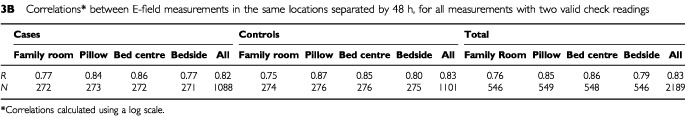
Correlations^*^ between E-field measurements in the same locations separated by 48 h, for all measurements with two valid check readings

shows the correlations between these repeated spot measurements. These suggest that E-field levels are stable from day-to-day, which is corroborated by the temporal stability of the 48-h measurement trace.

The UKCCS as a whole was designed as a matched case–control study, with the EMF component having measurements on one control per case. For the electric field sub-study, however, a substantial proportion of measurements belonged to participants who were paired to a subject with missing data. This was largely because the paired measurement did not have two valid check readings, though occasionally phase II measurements without electric field readings were carried out on one of a matched pair. To avoid loss of information, we therefore ignored the matching and used unconditional logistic regression to estimate the risk, adjusting for the matching variables in the form of age in years, sex and UKCCS region. All controls were used in each analysis. Previously, we found that socio-economic levels varied between cases and controls participating in the UKCCS as a whole (UKCCS Investigators, 2000b). We therefore included as a measure of socio-economic status the seven-level deprivation index derived from small-area census data used in our analysis of measured magnetic fields (UKCCS Investigators, 1999). This was based on unemployment, overcrowding and car ownership. The radon results from the UKCCS (UKCCS Investigators, 2002) indicate that some biases are present in the UKCCS data that cannot be accounted for by the available data on deprivation. However, since the factors affecting radon levels (open windows, floor level, underlying geological formation, etc.) would appear to be of little relevance to electric field strengths, it cannot be assumed that the biases in the radon results would be applicable to the present findings.

## RESULTS

Electric field measurements were made in the homes of 926 subjects. Table 1 shows the result of the check readings for these measurements. There were 549 subjects (273 cases, 276 controls) with E-field measurements containing two valid check readings, 75 (36 cases, 39 controls) of which were unmatched. Approximately one third of residential ELF electric field assessments had no check readings.

The mean time between diagnosis or pseudo-diagnosis and the initial measurement was 37.8 months (s.d. 14.4) for cases and also 37.8 months (s.d. 14.4) for controls, for the 549 subjects with acceptable instrument function checks. This was longer than the corresponding intervals of 20.8 months and 21.3 months to the initial measurements of cases and controls respectively in the complete magnetic field study (UKCCS Investigators, 1999), as the electric field pilot study was part of the second stage of measurements (phase II). Although the study methods meant that more residentially mobile families were less likely to be measured, there was no evidence that the controls included in the E-field study were less residentially stable than cases; for E-field participants with validated check measurements, the mean time that the family had lived in the measured home at (pseudo) diagnosis was 6.9 years (s.d. 4.5) for case families and 7.5 years (s.d. 5.1) for control families.

Table 4

**Table 4:**
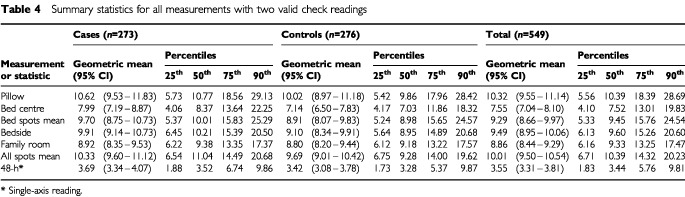
Summary statistics for all measurements with two valid check readings

shows the geometric mean (with 95% confidence interval) and 25th, 50th, 75th and 90th percentiles for the four spot measurements, the 48-h bedside measurement and the mean of the bed spots and the mean of all four spots, for all measurements with two valid check readings.

Table 5

**Table 5:**
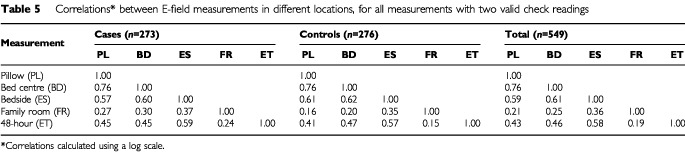
Correlations* between E-field measurements in different locations, for all measurements with two valid check readings

gives the correlations between E-field measurements in different locations. These are lower than the correlations between repeat measurements in the same location (Table 3b).

**Figure 2:**
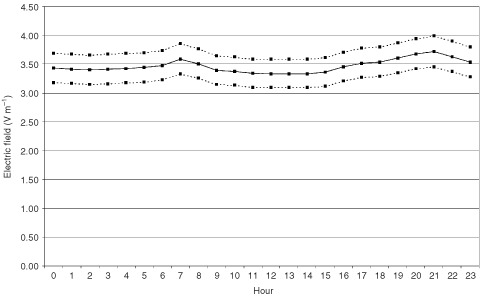
Hourly geometric mean E-field levels measured on a single vertical axis, with 95% confidence intervals, for readings with two valid check measurements (*n*=549).

The pairs of spot measurements in the same location were made at the same time of day, as they were either side of the 48-h measurement, and would therefore not reflect diurnal variation. The 48-h measurement of the vertical E-field component can be used to assess temporal stability over the day. Figure 2

shows the geometric means of the individual hourly averages, for all readings with valid check measurements. The average E-field levels vary little over the day, at least in their vertical component. There are small peaks in the morning and evening, perhaps due to increased appliance use when family members are getting up or have returned home for the evening from work and school.

The UKCCS as a whole included 87% of all eligible cases diagnosed in Great Britain in the period of interest and had a corresponding participation rate in controls of 64%, with some evidence of under-representation of controls resident in the most deprived census areas (UKCCS Investigators, 2000b). For subjects included in the EMF part of the study, the most deprived category was substantially under-represented, compared to the full set of potential first-choice controls, though there was little difference in the deprivation distribution between the cases and controls (UKCCS Investigators, 1999). Table 6

**Table 6:**
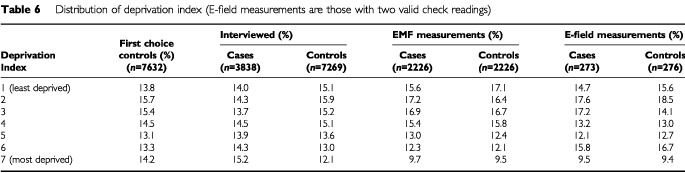
Distribution of deprivation index (E-field measurements are those with two valid check readings)

gives the census-based small-area deprivation indices for the following groups: the complete set of first choice controls; interviewed cases and controls; cases and controls included in the EMF study; the subset of the cases and controls in the EMF study with E-field measurements. Allowing for the smaller numbers, the deprivation distribution in subjects with E-fields measurements is similar to the EMF study group overall.

The distributions of age, sex and deprivation index for each diagnostic category are given in Table 7

**Table 7:**
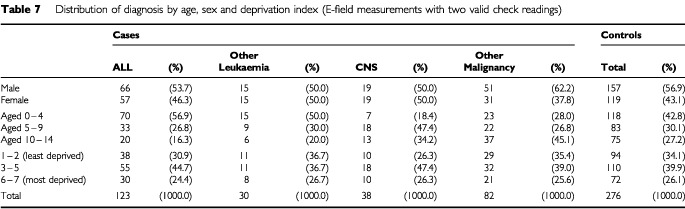
Distribution of diagnosis by age, sex and deprivation index (E-field measurements with two valid check readings)

. The results are given for groups of age and deprivation index, as the more detailed table would be sparse. The deprivation index distribution is similar in the control group and the different diagnostic categories.

Table 8

**Table 8:**
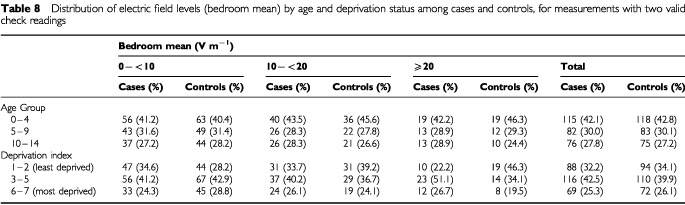
Distribution of electric field levels (bedroom mean) by age and deprivation status among cases and controls, for measurements with two valid check readings

presents the distribution of exposure (mean of bed measurements), categorised as described in the statistical methods section, for age and deprivation index groups among the cases and controls. E-field levels appear not to be age related, but there is some suggestion that they are associated with deprivation.

We examined the risk associated with different levels of electric field exposure, measured by the mean of the bed measurements, for acute lymphoblastic leukaemia (ALL), all leukaemias, central nervous system tumours, other malignancies and all malignancies (Table 8a and b). For the primary results, based on measurements with two valid check readings (Table 9a

**Table 9a:**
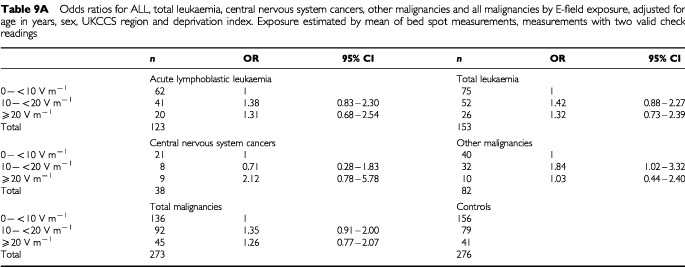
Odds ratios for ALL, total leukaemia, central nervous system cancers, other malignancies and all malignancies by E-field exposure, adjusted for age in years, sex, UKCCS region and deprivation index. Exposure estimated by mean of bed spot measurements, measurements with two valid check readings

), there is a slightly elevated but non-significant risk for children with exposures of 10 to 20 V m^−1^ and more than 20 V m^−1^, when compared to the baseline group with exposures of less than 10 V m^−1^. For acute lymphoblastic leukaemia, the odds ratio for the comparison between exposures of greater than 20 V m^−1^ versus those less than 10 V m^−1^, is 1.31, with a 95% confidence interval of 0.68 to 2.54, after adjustment for matching variables and the index of deprivation. For all malignancies combined, the situation is similar, with slightly narrower confidence bounds due to greater numbers. For malignancies not otherwise classified, an adjusted odds ratio of 1.84 (1.02–3.32) was obtained for children with mean exposures of 10 to 20 V m^−1^. This was the only odds ratio differing significantly from unity. The corresponding value for subjects with mean exposure greater than 20 V m^−1^, however, was 1.03 (0.44–2.40), and the results overall show little evidence of increasing risk with increasing dose. When the larger sample containing unvalidated measurements is used (Table 9b

**Table 9b:**
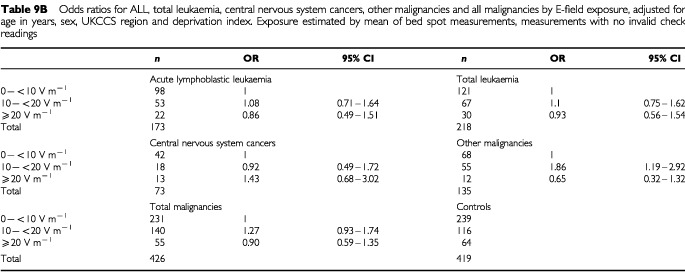
Odds ratios for ALL, total leukaemia, central nervous system cancers, other malignancies and all malignancies by E-field exposure, adjusted for age in years, sex, UKCCS region and deprivation index. Exposure estimated by mean of bed spot measurements, measurements with no invalid check readings

), the risk estimates are generally lower. For ALL, the adjusted odds ratio for the comparison between exposures of greater than 20 V m^−1^ and the baseline category becomes 0.86 (0.49–1.51).

The results of fitting electric field exposure as a continuous variable are presented in Table 10

**Table 10:**
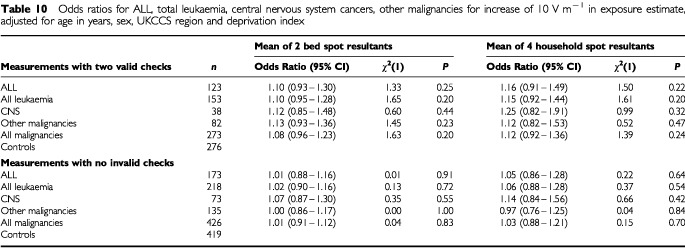
Odds ratios for ALL, total leukaemia, central nervous system cancers, other malignancies for increase of 10 V m^−1^ in exposure estimate, adjusted for age in years, sex, UKCCS region and deprivation index

. Odds ratios are given for an increase of 10 V m^−1^ for both the mean of the two bed spot resultants and the mean of the four household spot resultants. None of the odds ratios obtained is significantly different from one, though the ratios are greater than one for most diagnostic categories. As for the categorical analysis, the risk estimates are lower when the larger sample including unvalidated measurements is used. Here, the odds ratios are very close to one.

Unperturbed electric field strengths from high voltage overhead power lines were assessed for all subjects for whom we had details of sources of electricity supply near the home (3380 cases, 3390 controls). These were analysed using the same categories (<10 V m^−1^, 10–<20 V m^−1^, ⩾20 V m^−1^) as the analysis of measured fields (table not shown), with *n*=(3333, 17, 30) for all cases and *n*=(3347, 17, 26) for controls. None of the risk estimates obtained were significant. For the ⩾20 V m^−1^ category, odds ratios of 1.12 (95% CI 0.58–2.17) were obtained for total leukaemia, based on 14 cases in the highest category, and 1.18 (95% CI 0.69–2.00) for all malignancies.

## DISCUSSION

This investigation should be regarded a pilot study, since it was added on halfway through the main UKCCS and included only a small proportion of UKCCS cases. Nevertheless, as the literature on the epidemiology of exposure to ELF electric fields is sparse, our present study adds materially to a small body of knowledge both of exposure levels in the population and in the association with childhood malignancy. A survey of 40 homes by the National Grid Company (Swanson, 1999) found that background residential electric field levels, away from appliances, generally lie in the range 0–20 V m^−1^. Mean levels at the centres of rooms were 13.2 V m^−1^ with lights on and 10.4 V m^−1^ with lights off. Levels on beds had a mean of 14.5 V m^−1^. These values are arithmetic means. In our study, the arithmetic means for the combined bed measurements were 13.0 V m^−1^ (s.d. 14.2) and 10.5 V m^−1^ (s.d. 13.1) for the measurements made in the centre of the family room. A small number of studies outside the UK have measured residential ELF electric fields. A report from New Zealand, which has a similar electricity distribution voltage to the UK (230 volts), gave geometric means of 3.1 and 8.3 V m^−1^ for controls' daytime rooms and bedrooms respectively, based on readings from a single axis instrument for a sample of 117 homes (Dockerty *et al*, 1998). A study from the US, where the distribution voltage is 110 V, has reported ‘high power’ (appliances on) mean resultant levels ranging from 8.7 to 11.5 V m^−1^ across five categories of home classified by the US wire code system (*n*=278) (Savitz *et al*, 1988). Other studies have reported similar mean levels: 8 V m^−1^ at the centre of control children's bedrooms (*n*=108) (London *et al*, 1991), and 8 V m^−1^ for a combination of measurements at the centre of kitchen, bedroom and family room (*n*=42) (Kaune *et al*, 1987).

The E-field sample was drawn from phase II of the UKCCS magnetic field study, and so contains a disproportionate number of homes with higher magnetic field levels. The correlations between residential magnetic and electric field levels at the same time and location are weak (*r*=−0.01 to 0.14 on a log scale for spot measurements in the different locations, for the 549 validated readings). This absence of correlation means that selecting on magnetic field levels implies negligible selection on electric field levels.

We have made measurements at individual residential locations, repeated after a period of 48 h, with correlations over time (on a log scale) between 0.76 and 0.86, implying temporal stability (Table 3b). This contrasts with the large spatial variation demonstrated by the low correlation between the levels downstairs at the centre of the family room and those upstairs in the child's bedroom (*r*=0.21–0.36 for log transformed data, cases and controls, Table 5). Indeed, only moderate correlation was seen between bedside measurements and those on the bed, either on the pillow or the bed centre (*r*=0.59–0.61 for log transformed data, cases and controls, Table 5). These findings are indicative of the local E-field environment being determined by local sources. Electric field levels found on the bed are higher than at the centre of room (Table 4), which could be due to the increased proximity of local sources, such as wiring and appliances, or to perturbation of the field by the bed. We found that the distribution of the family room centre measurements was more symmetrical and less skewed than that of the measurements made on the child's bed.

With the residential E-field environment being more spatially variable than the magnetic field, use of particular household measurements as a surrogate for average exposure is problematic. However, young children spend around half their time in bed (from questionnaire data, we found that children aged 0 to 5 have sleeping time of over 11 h, on average). We have completely characterised the pillow and bed centre locations through measurement of three orthogonal components of the unperturbed E-field. The root-sum-squared (RSS) resultant field at these two locations shows moderately strong correlation, (*r*=0.76, Table 5) and stability over time at both locations is indicated through strong correlation of spot measurements separated by 48 h. The bedside 48-h measurement provides an index of temporal stability through the logging of the vertical E-field component. There is also a strong correlation between the vertical component of the bed spot measurements and the average bedside 48-h vertical component, *r*>0.8. Our pillow and bed measurements would therefore appear to characterise adequately the fields that a child will encounter when going to bed. The presence of the child will perturb the field to a degree determined by factors such as the child's posture and the field direction. Since bed measurements are more variable than the family room or centre of bedroom measurements, we consider that use of the two bed measurements would capture much of the inter-individual variation in exposure.

We found no association between E-field exposure assessed through bedroom measurements and childhood cancer. For acute lymphoblastic leukaemia, for total leukaemia and for CNS cancers, odds ratios did not differ significantly from unity and there was little indication of a dose–response (Table 10). For malignancies other than leukaemia or CNS cancers, although exposure in the intermediate {⩾10 and <20} V m^−1^ category was associated with a significantly elevated odds ratio of 1.84, the odds ratio for higher exposure did not differ significantly from unity. Moreover, there was no indication of a dose response in this disease category (Table 10). When electric field exposure was modelled as a continuous variable, odds ratios using the principal metric were close to unity for all disease categories, never differing significantly from one.

Our failure to find evidence of increased risk accords with other major studies of childhood electric field exposure. Two earlier American epidemiological studies into childhood E-field exposure (Savitz *et al*, 1988; London *et al*, 1991) found no significant elevation of risk with centre room spot measurements as exposure metrics. The authors, however, did appreciate the uncertainty in estimating exposure with the metric available. A study from New Zealand (Dockerty *et al*, 1998) found no significant increase in risk using as exposure metrics the means of two 24-h measurements made in the centre of the most used room and by the child's bed. Two recent Canadian studies (Mcbride *et al*, 1999; Green *et al*, 1999) found no increase in risk using 48-h personal monitoring. The influence of the body on the electric field during personal monitoring, together with the possibility that case children may have changed their behaviour since developing the disease make these results hard to interpret. Some studies of occupational E-field exposures in adults have suggested an increased risk of leukaemia and brain tumours in jobs with high exposures to electromagnetic fields, but a recent authoritative review (Iarc, 2002) concluded that there was inadequate evidence for the carcinogenicity of extremely-low frequency electric fields.

One report has suggested that elevated E-field exposure could augment risk of childhood cancer. Researchers from the UK (Coghill *et al*, 1996), using the vertical component of E-field on and around the bed, reported a significantly elevated risk of childhood cancer of 4.7 (95% CI 1.17–27.78). In that study, where diagnosis was unconfirmed and the unblind exposure metric was from inconsistent locations, there was the suggestion of a log normal distribution of measured bedroom levels in the control population. The shape of the distribution was strikingly different in the case population, with a flat distribution across elevated exposure categories. No such difference in measurement distribution is evident in our present study. To investigate the possibility of vertical component alone increasing risk, we have used the vertical component of pillow and centre bed measurements as exposure metrics in unconditional regression analysis. Again the results for vertical component showed no significant elevation of risk for any disease category examined. The sample size of our study, although small for the detection of a small risk, would be sufficient to uncover a risk of the level suggested by the above report.

We have also examined two other sources of potential E-field exposure associated with a child's residence. The first is exposure from appliances with the potential to materially influence average E-field exposure, predominantly electric blankets, though night storage heaters are also included. In the absence of detailed information on the range of exposure associated with the use of these appliances, a subsidiary analysis has been undertaken with particular appliance use as additional categorical variables. Of the 549 individuals (276 cases, 273 controls) with two valid check readings, 44 (22 cases, 22 controls) had night storage heaters and eight (five cases and three controls) used electric blankets. This analysis showed no evidence of any significant increase in risk associated with the use of these appliances. This finding is in some contrast to two recent studies finding some evidence of increased leukaemia risk being associated with children's use of electric blankets; the first reporting an odds ratio of 2.75 (95% CI 1.52, 4.98) (Hatch *et al*, 1998) and the second citing an odds ratio of 2.2 (95% CI 0.7, 6.4) (Dockerty *et al*, 1998). None of our leukaemia cases with E-field measurements used electric blankets.

In addition to appliance use, we have also considered unperturbed E-fields external to the residence generated by nearby overhead high voltage electricity circuits. High voltage power lines produce some of the highest power-frequency electric field strengths encountered, up to 11 kV m^−1^ directly underneath 400 kV lines. In the present study, external field strengths of up to 1.6 kV m^−1^ have been calculated. However, the relevance of external fields in the estimation of exposure is uncertain given the strong attenuation of ELF E-fields by conducting materials such as the fabric of buildings and vegetation. We found only weak correlations between external calculated E-field levels and internal measured levels (*r*=0.05 for the mean of all the spot measurements, *r*=0.08 for the mean of the bed spot measurements, using log-transformed data, for the 47 subjects with validated measurements who lived sufficiently close to a line to have a field calculated). Furthermore, the proportion of a child's life spent at locations where the E-field environment is influenced by external sources is unknown and likely to be highly variable. Nevertheless, we have analysed the unperturbed external field at the residence, as an individual factor for risk. The results are consistent with the analysis of measured E-fields with no significant increase in risk and no suggestion of a dose response being uncovered. This is unsurprising in that close to power lines calculated E-fields are well correlated with calculated magnetic fields, (*r*=0.76 for log transformed data, *n*=234) which were not associated with disease in our previous analysis (UKCCS Investigators, 2000a). The only other study to investigate childhood cancer and calculated electric fields known to us (Tynes and Haldorsen, 1997) did not report exact results, but noted that ‘electric fields were not significantly associated with cancer’.

In summary, this pilot study provides no support for the hypothesis that residential exposure to ELF electric fields is associated with childhood cancer either by disease category or in total. The study can exclude electric field exposure as a cause of a substantial proportion of leukaemia or other childhood malignancies in the UK. Efforts to uncover the causes of childhood malignancy appear better targeted in other directions.

## References

[bib1] CoghillRWStewardJPhilipsA1996 Extra low frequency electric and magnetic fields in the bedplace of children diagnosed with leukaemia: a case-control study Eur J Cancer Prev 55153158 8818603 10.1097/00008469-199606000-00002

[bib2] DockertyJDElwoodJMSkeggDCGHerbisonGP1998 Electromagnetic field exposures and childhood cancers in New Zealand Cancer Causes Control 93299309 9684710 10.1023/a:1008825220759

[bib3] GreenLMMillerABVilleneuvePJAgnewDAGreenbergMLLiJDonnellyKE1999 A case–control study of childhood leukemia in southern Ontario, Canada, and exposure to magnetic fields in residences Int J Cancer 822161170 10389746 10.1002/(sici)1097-0215(19990719)82:2<161::aid-ijc2>3.0.co;2-x

[bib4] HatchEELinetMSKleinermanRATaroneRESeversonRKHartsockCTHainesCKauneWTFriedmanDRobisonLLWacholderS1998 Association between childhood acute lymphoblastic leukemia and use of electrical appliances during pregnancy and childhood Epidemiology 93234245 9583414

[bib5] IARC2002 IARC monographs on the evaluation of carcinogenic risks to humans, Vol. 80, Non-ionizing radiation, Part 1: Static and extremely low-frequency (ELF) electric and magnetic fieldsLyon: IARC PMC5098132 12071196

[bib6] KauneWTStevensRGCallahanNJSeversonRKThomasDB1987 Residential magnetic and electric fields Bioelectromagnetics 84315335 3426634 10.1002/bem.2250080402

[bib7] LondonSJThomasDCBowmanJDSobelEChengT-CPetersJM1991 Exposure to residential electric and magnetic fields and risk of childhood leukemia Am J Epidemiol 1349923937 1843457 10.1093/oxfordjournals.aje.a116176

[bib8] McBrideMLGallagherRPThériaultGArmstrongBGTamaroSSpinelliJJDeadmanJEFinchamSRobsonDChoiW1999 Power-frequency electric and magnetic fields and risk of childhood leukemia in Canada Am J Epidemiol 1499831842 10221320 10.1093/oxfordjournals.aje.a009899

[bib9] SavitzDAWachtelHBarnesFAJohnEMTvrdikJG1988 Case-control study of childhood cancer and exposure to 60-Hz magnetic fields Am J Epidemiol 12812138 3164167 10.1093/oxfordjournals.aje.a114943

[bib10] SwansonJ1999 Residential power-frequency electric and magnetic fields: sources and exposures Radiat Prot Dosim 831–2914

[bib11] TynesTHaldorsenT1997 Electromagnetic fields and cancer in children residing near Norwegian high-voltage power lines Am J Epidemiol 1453219226 9012594 10.1093/oxfordjournals.aje.a009094

[bib12] UK Childhood Cancer Study Investigators1999 Exposure to power-frequency magnetic fields and the risk of childhood cancer Lancet 354919419251931 10622294

[bib13] UK Childhood Cancer Study Investigators2000a Childhood cancer and residential proximity to power lines Br J Cancer 831115731580 11076671 10.1054/bjoc.2000.1550 PMC2363427

[bib14] UK Childhood Cancer Study Investigators2000b The United Kingdom Childhood Cancer Study: objectives, materials and methods Br J Cancer 82510731102 10737392 10.1054/bjoc.1999.1045 PMC2374433

[bib15] UK Childhood Cancer Study Investigators2002 The United Kingdom Childhood Cancer Study of exposure to domestic sources of ionising radiation: 1: radon gas Br J Cancer 861117211726 12087456 10.1038/sj.bjc.6600276 PMC2375400

